# Understanding the “black box” of a health-promotion program: Keys to enable health among older persons aging in the context of migration

**DOI:** 10.3402/qhw.v10.29013

**Published:** 2015-12-08

**Authors:** Emmelie Barenfeld, Susanne Gustafsson, Lars Wallin, Synneve Dahlin-Ivanoff

**Affiliations:** 1 Department of Health and Rehabilitation, Institute of Neuroscience and Physiology, The Sahlgrenska Academy, University of Gothenburg, Gothenburg, Sweden; 2 Department of Occupational Therapy and Physiotherapy, The Sahlgrenska University Hospital, Gothenburg, Sweden; 3 Centre for Ageing and Health – AgeCap, University of Gothenburg, Gothenburg, Sweden; 4 School of Education, Health, and Social Studies, Dalarna University, Falun, Sweden; 5 Division of Nursing, Department of Neurobiology, Care Sciences and Society, Karolinska Institutet, Solna, Sweden

**Keywords:** Immigrant, emigrant, occupation, group intervention, person-centeredness, grounded theory, health-promoting messages

## Abstract

Although the need to make health services more accessible to persons who have migrated has been identified, knowledge about health-promotion programs (HPPs) from the perspective of older persons born abroad is lacking. This study explores the design experiences and content implemented in an adapted version of a group-based HPP developed in a researcher–community partnership. Fourteen persons aged 70–83 years or older who had migrated to Sweden from Finland or the Balkan Peninsula were included. A grounded theory approach guided the data collection and analysis. The findings showed how participants and personnel jointly helped raise awareness. The participants experienced three key processes that could open doors to awareness: enabling community, providing opportunities to understand and be understood, and confirming human values and abilities. Depending on how the HPP content and design are being shaped by the group, the key processes could both inhibit or encourage opening doors to awareness. Therefore, this study provides key insights into how to enable health by deepening the understanding of how the exchange of health-promoting messages is experienced to be facilitated or hindered. This study adds to the scientific knowledge base of how the design and content of HPP may support and recognize the capabilities of persons aging in the context of migration.

The need to make health services more accessible to persons who have migrated by addressing barriers in service provision and considering the experience of health-related beliefs has been previously identified (Alizadeh-Khoei, Mathews, & Hossain, 2011; Aroian, Wu, & Tran, 2005; Fassaert, Hesselink, & Verhoeff, 2009; Pitkin Derose, Bahney, Lurie, & Escarce, 2009; Rechel, Mladovsky, Ingleby, Mackenbach, & McKee, 2013). However, there is still a need to evaluate the approaches used to improve health service provision to persons who have migrated, specifically, adapted health-promotion programs (HPPs) (Liu et al., 2012; Rechel et al., 2013). Therefore, this study will focus on the experiences of an adapted version (Gustafsson et al., 2015) of an evidence-based HPP (Dahlin-Ivanoff et al., 2010) from the perspective of older persons aging in the context of migration.

The proportion of people aging outside their country of birth is increasing in many European countries, including Sweden (Rechel et al., 2013). In Sweden, approximately 12% of all people aged 65 years or older were born abroad (Statistics Sweden, 2012). This means that more than one out of every 10 people is aging in the context of migration; that is, they have migrated from their country of birth to reside in another country, where they are going through the aging process. Migration can be seen as a social determinant of health (Marmot et al., 2012) and may influence how older persons experience health during the aging process (Kulla, Ekman, & Sarvimäki, 2010; Torres, 2001). Aging is also associated with an increased risk of health problems (Femia, Zarit, & Johansson, 2001; Smith, Borchelt, Maier, & Jopp, 2002). For these reasons, older persons born abroad are a priority population for health promotion.

Health promotion is defined as the process of enabling people to increase their control and improvement of their health. Health encompasses more than just the absence of disease, referring to both personal and social resources as well as physical capabilities (WHO, 1998). Health and occupation are interrelated, and enabling older people to be engaged in meaningful occupations is considered important (Townsend & Polatajko, 2007; Wilcock, 2007). Enablement is considered one of the key strategies for health promotion and refers to ensuring equal opportunities and resources so that all people can achieve their fullest health potential (WHO, 1986). However, previous research indicates that migration may result in the loss of factors that enable persons to maintain daily activities and health (Alizadeh-Khoei et al., 2011; Bennett, Scornaiencki, Brzozowski, Denis, & Magalhaes, 2012; Pitkin Derose et al., 2009; Torres, 2001). HPPs may be one strategy to enable health in the aging population (WHO, 2009). Currently, there is a lack of studies exploring older foreign-born persons’ experiences of HPPs aimed at supporting their independence in everyday activities.

The Promoting Aging Migrants’ Capabilities study (Gustafsson et al., 2015) is evaluating an HPP targeting persons aged 70 years or older who have migrated to Sweden from Finland or the Balkan Peninsula. The goal of the program is to facilitate access to knowledge of the aging process and possible strategies to manage problems that may occur in everyday life among older people aging in the context of migration. The program contains both group meetings and an individual follow-up home visit. It is administrated by an interprofessional team with a person-centered approach to health promotion (Gustafsson et al., 2015). This approach encourages all health-promoting decisions to be taken in partnership (Ekman et al., 2011).

Multidimensional programs, such as the Promoting Aging Migrants’ Capabilities study (Gustafsson et al., 2015), are complex by nature. This means that several program components may interact and open up a range of possible outcomes (Craig et al., 2013), which poses challenges for their evaluation (Jolley, 2014). Further, there are no guarantees that program components will be implemented as intended (Craig et al., 2013). Therefore, it is important to deepen the understanding of which health-promoting processes experienced by older persons born abroad were implemented during the HPP and under which circumstances these processes occurred. The exploration of this kind of knowledge is referred to as “unpacking the ‘black box’ of a program” (Astbury & Leeuw, 2010) and is requested in studies about health promotion for older people (Beswick et al., 2008; Clark et al., 2012). Thus, to improve our understanding of program outcomes and support future program development, this study aimed to explore the experiences of the implemented content and design of a HPP among persons aging in the context of migration.

## Method

### Design and study context

A grounded theory design developed by Charmaz (2006) was used because it is considered suitable for studying processes and actions. According to Charmaz (2006), grounded theory is emphasized to contribute to understanding rather than providing explanation, thus fulfilling the goal of this study.

This study is a part of a larger collaborative project, the Promoting Aging Migrants’ Capabilities study (Gustafsson et al., 2015). It took place in an urban district situated outside the city center, but within the city limits of a medium-sized city in western Sweden. Fifty percent of all inhabitants in the urban district were born in countries outside Sweden. Of the total population, 11% were 65 years or older. For people aged 65 years or older who were born abroad, Finland, Bosnia-Herzegovina, and the former Yugoslavia were the main countries of birth. For more details, see Gustafsson et al. (2015).

### The Promoting Aging Migrants’ Capabilities program

The HPP in this study, the Promoting Aging Migrants’ Capabilities program, comprised four weekly senior group meetings attended by an interprofessional team and an individual follow-up home visit. The home visit was performed by one professional, whereas the interprofessional team, where the team members were responsible for one session each, administrated the group meetings. The interprofessional team consisted of an occupational therapist, a physiotherapist, a registered nurse, and a qualified social worker. In addition, one of the team members followed each set of the senior meetings and served as a group leader. Group meetings were used to enable participants to learn from each other, through peer learning (Shiner, 1999). A booklet covering different aspects of self-management of health (such as physical activity, medication, nutrition, assistive devices, adaptation of housing, memory, and quality of life) served as a basis for group discussions. Therefore, it was recommended that participants prepare for each session by reading the booklet or listening to the audio book. The booklet themes and the professionals responsible for each session are shown in Table I. The content and discussions during the senior group meetings varied according to the participants’ experiences, needs, and resources. This design was in line with the person-centered approach, which highlights people's expertise regarding their own situation (Leplege et al., 2007).

**Table I:** Booklet themes.

Booklet themes	Principal professional^a^
Aging	PT
Physical activity helps keep you physically fit	PT
Food is a prerequisite for health	PT
You can take care of problems with your health	RN
How to use medicine	RN
Coping with everyday life	OT
You do not need to feel insecure	OT
Technology in everyday life	OT
Will I lose my memory?	OT
Life events and quality of life during aging	SW
Anyone who needs help can get help	SW

a PT, physiotherapist; RN, registered nurse; OT, occupational therapist; SW, social worker.

The HPP was developed in a researcher–community partnership based on an evidence-based intervention, Elderly Persons in the Risk Zone (Dahlin-Ivanoff et al., 2010). The adaptation from the original protocol to the current protocol has been described elsewhere (Gustafsson et al., 2015; Lood, Gustafsson, & Dahlin-Ivanoff, 2015). A bilingual approach was adopted: interpreters were available during the meetings, and written materials were printed both in Swedish and in the participants’ mother tongue. New information was added to the booklet concerning how to handle posttraumatic stress in everyday life. In summary, the design and content of the HPP were characterized by several components: multidimensional health information, interprofessional teamwork and provision, person-centered approach, peer learning, language modifications, and assessments of function and home environment (Gustafsson et al., 2015; Lood, Gustafsson et al., 2015).

### Sampling and participants

The sample was recruited from the HPP (Gustafsson et al., 2015), which took place between 2012 and 2014. The HPP included persons aged 70 years or older who were independent in their daily activities, lived in ordinary housing in the urban district, and who had migrated to Sweden from either Finland or the Balkan Peninsula. The single exclusion criterion was impaired cognition, defined as below 80% of the administered items on the mini-mental state examination (Folstein, Folstein, & McHugh, 1975). For this study, persons who had participated in the HPP were selected by purposeful sampling according to initial sampling criteria that strove for heterogeneity in age, gender, type of housing, marital status, and spoken language during the senior group meetings. Participants were enrolled between December 2012 and March 2015 until theoretical saturation was reached (Charmaz, 2006). The participants represented six different senior meeting groups of varying size. An interpreter was available during the meetings for eight of the interviewed participants. All persons who were asked consented to participate in the study.

In total, 14 participants aged 70–83 years were included: eight women and six men. Nine participants had migrated from Finland and five from the Balkan Peninsula. All had been living in Sweden for at least 11 years and most for 21 years or more. Half of the participants had migrated for work- or education-related reasons and the rest for family reasons (*n*=3) or to find safe refuge (*n*=3). Participant characteristics are shown in Table II.

**Table II:** Participant characteristics.

Characteristics	Number (*n*=14)
Age (years)	
70–75	7
76–80	5
Over 80	2
Marital status	
Married/cohabiting	8
Widowed/single living	6
Type of housing	
Owner of house or apartment	8
Tenant	6
Educational status (more high school graduation)	10
Health self-estimated as *good* a or better	8
Language skills and preferences	
Experience difficulties or inability to make oneself understood	5
Prefer to speak mother tongue during the HPP	5
Interpreter available during the HPP	8

a Question from the 36-item Short Form Health Survey. Responses were graded on a 5-point Likert scale: *bad*, *fairly good*, *good*, *very good*, and *excellent*. HPP, health-promotion program.

### Ethical considerations

The study followed the ethical principles of the Declaration of Helsinki and was approved by the regional ethics review board on December 13, 2012 (registration number T947-12).

There were several ethical issues connected to language because the goal was to include persons with varied Swedish language skills. For instance, written and oral information for informed consent was given in the participants’ preferred language to ensure that they were aware of what participation entailed and that they could terminate their participation at any time. There is always a risk that interviews will provoke negative emotions, which must be weighed against the possible benefits of the research project. In anticipation of any emotional reactions or other questions, the interviewers allocated extra time for the interviews. The interviewers were also sensitive to signs of fatigue and breaks were taken when needed.

### Data collection

Individual in-depth interviews were conducted in the homes of the participants and lasted 77 min on average (range 18–167 min). Nine participants choose to be interviewed in Swedish. These interviews were conducted by the first author. Interviews performed in the participants’ mother tongues were conducted by research assistants who were university educated and fluent in both Swedish and the required language. In two of the interviews, a family member accompanied the participant for moral support in accordance with the participant's wishes. The interviews were tape-recorded and transcribed verbatim in Swedish by the first author or in the mother tongue by the research assistants, who then also translated the interviews into Swedish.

An interview guide was used to facilitate the interviews and covered question areas concerning the design and content of the HPP, such as the booklet, four group meetings, follow-up home visit, group discussions, personnel and interpreter, person-centeredness, time, and design. In line with the theoretical sampling, the interview guide was complemented with new question areas according to the ongoing analysis (Draucker, Martsolf, Ross, & Rusk, 2007). To cover the process from the first experience of the HPP, the interview guide started with the question, “Can you tell me about your thoughts when you received the invitation letter?” Follow-up questions were asked, such as, “Can you tell me more about that?” or “What does it mean to you?”

### Data analysis

Data collection and analysis were done simultaneously (Charmaz, 2006). Throughout the process, all data were systematically compared with all other data through constant comparison (Charmaz, 2006; Hallberg, 2006). Therefore, coding, memo writing, and constant comparisons started with the first interview and were an ongoing iterative process through the analysis.

Charmaz's (2006) descriptions of initial and focused coding were used. First, each line was coded in close connection to the data with openness to explore theoretical possibilities discerned in the data (initial coding). If possible, these codes were formulated as gerunds to support the identification of action in each segment of data. Memos were written to record what was happening during data collection, and comparisons were made of data both within and between interviews. Later on, focused coding was used to sift through larger amounts of data by using the most significant and frequent initial codes. Then segments of data were synthesized and explained with a conceptual code. All codes were compared and sorted into categories. In this process, memo writing was used to compare data systematically and describe how categories emerged (Charmaz, 2006). To stay true to the essence of the data, the first author performed the analysis in Swedish. The research assistants wrote memos after each interview, verified the essence of the coding of their conducted interviews, and participated in discussions during the analysis to minimize barriers related to translation.

## Results

### Opening doors jointly to awareness of past, present, and later life

**Figure 1:**
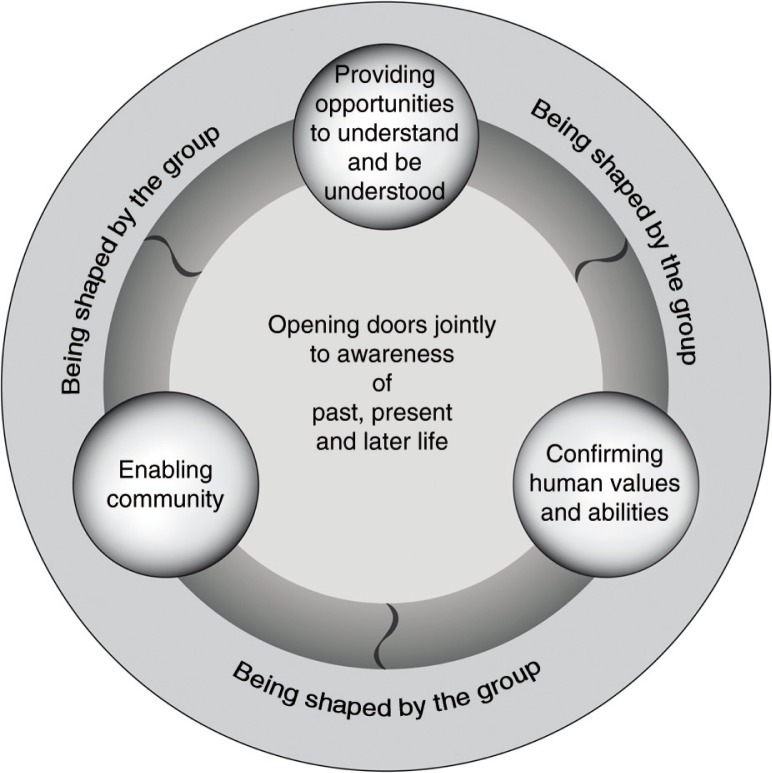
Model visualizing the understanding of the “black box” of a health-promotion program from the perspective of persons aging in the context of migration.

Our analysis identified the core category *opening doors jointly to awareness about past, present, and later life*. This category highlights the experience of gaining deeper insight into one's own or another person's past, present, or later life, by considering or doing things one usually does not do. The experience of awareness varies from person to person both in depth and content. What a person becomes aware of is influenced by which doors to awareness the HPP is opening, whereas the level of awareness is influenced by how the doors are opened and for how long. Three key processes open doors to awareness: *enabling community*, *providing opportunities to understand and be understood*, and *confirming human values and abilities*. Each of these three key processes is *being shaped by the group* and concerns the past, present, and later life (see Fig. 1), which means that they could be more or less present depending on how the HPP content and design is being shaped by the group.

### Being shaped by the group

The group acts as a filter that shapes the processes that are used to open doors to awareness and determines how these processes are experienced. Each group session is experienced as being shaped by the group, which means that each session is different because the three key processes are targeted in various amounts and at different depths. Everyone, that is, the participants, personnel, and interpreters, is described to influence the provision of health-promoting actions and messages and thereby shape the key processes.

The group both stimulate and inhibit the key processes to opening doors to awareness, according to how the HPP content and design is being shaped by the group. The people involved contribute to this process in different ways. The participants experience the personnel as having a leading role in ensuring that group conversations become balanced and that the sessions are kept to their allocated time. They also experience that personnel stimulate their responsibility to act by using an approach that opens up conversations and reflections and creates an arena that provides the opportunity to make choices. Therefore, participants experience that they can contribute their own experiences, search for information according to their needs and interests, and regulate the depth of the conversations.

### Enabling community

The key process *enabling community* refers to the social value of seeing and socializing with peers and personnel during the baseline assessment, senior group meetings, or the follow-up home visit. Meeting others gave participants the opportunity to have conversations about their current life situation, historical life events, and possible future scenarios. In this way, the key process contributed to the opening of doors to awareness about past, present, and later life. Sometimes the main reason to participate was the opportunity to get together with peers who shared the experience of aging or had experienced similar historical events. If there was a lack of social venues after the HPP, the participants often stopped seeing each other even if they expressed a wish to continue:

This was a sort of safety valve for us seniors. At this age, we don't get to meet a lot of people anymore. Earlier, when we were working in our more active years, we met workmates – we had to. But now we're more confined. Now (because of HPP), one is able to get out and meet others, talk a bit, let off steam, reduce pressure, the inside pressure. (Participant 12)

Depending on how the key process *enabling community* is shaped by the group, participants may experience both a sense of belonging and of alienation, which may occur in the same person and may impact the ability to connect to other group participants. Therefore, the sense of belonging or feeling of alienation shapes the experience of community. Activities such as sharing experiences and stories about ones lives’ mostly contribute to experiences of growing together and feeling like a group. However, sharing experiences and stories may also contribute to feelings of alienation on occasion if the composition of group participants or topics of conversation make a person feel differently in relation to others. One person expressed this in relation to being invited to participate:

Yes, I suppose it was partly due to the fact that they were all 70 years old, and that I was so much older. And then I thought they were the sort that played golf and [coughs] went fishing and [did] all sorts of things. And I fished too. At any rate, I did when I was 60. Then I thought, yes, I actually thought that they should've taken someone else rather than me. (Participant 5)

### Providing opportunities to understand and be understood

The key process, *providing opportunities to understand and be understood*, is characterized by the experience of getting prerequisites to search for and acquire knowledge for oneself or for other people, such as neighbors or relatives. The essence of providing opportunities to understand is twofold. First, it means feeling assured of understanding and being understood linguistically. Second, it refers to the experience of facilitating conditions for learning, which supports the understanding of opportunities available in one's everyday life and in relation to society. This key process opens doors to awareness when the HPP content and design brings new perspectives or serves as a reminder of what is important to know or do.

The senior group meetings have been enlightening and helped a lot with things that I had no idea about [short pause], and [have] given me opportunities. I had no idea of my rights and possibilities that can be used to live a better and safer everyday life. (Participant 10)

Opportunities to understand emerge because the variation in HPP design and content provides alternative ways of opening doors to awareness. This was experienced as having access to different learning styles, memory support, repetition, and the possibility to choose a source of information that participants could understand and depend on. Being offered choices of how to learn, what to learn, and by whom helped overcome barriers between participants’ functions and environmental demands. In addition, being able to use a language that had been mastered, such as Swedish or one's mother tongue, was experienced as a prerequisite to understanding. Mastery of language relates to both understanding others and making oneself understood. Some participants indicated the opportunity to use two languages as a necessary means of achieving mutual understanding. Furthermore, bilingual use during the HPP was experienced as improving future understanding by providing opportunities to practice Swedish language skills, which could improve the vocabulary for everyday conversations or for future contact with any health services.

I understood that it was good for me [to learn words used in terms of health care] that I knew, words such as *physiotherapist* and/or *podiatrist* and so on. One can get them mixed up. Okay, I'm probably thinking the right thing, but since I practice Swedish too little, I get words mixed up. And you know they mean completely different things. (Participant 11)

Depending on how the key process, *providing opportunities to understand and be understood*, is shaped by the group, there is variation in the way it opens doors to awareness and the things of which people become aware. The personnel support participants’ reflections along the concepts of past, present, and later life using the booklet content and in relation to the participants’ conversations and life stories. When participants are at different stages of the aging process in relation to each other, they also support this process because they shape the health-promoting messages by advising others and searching for information in accordance with their current needs, interests, and previous experiences. The interpreters and participants sharing the same mother tongue shape this process by assuring understanding.

### Confirming human values and abilities

The key process *confirming human values and abilities* is characterized by personnel, peer, or research team actions that support the experiences of being recognized as capable persons who are of value to society regardless of age or origin. This key process contributes to opening doors to awareness about past, present, and later life by targeting who participating persons were, are, or may be as well as what they did, do, or are supposed to be capable of doing.

*Confirming human values* means showing that each person is valued and can be depended on. This experience emerges through the ability to make one's voice heard and by recognizing, seeing, and listening to each other in the senior group meetings. In addition, receiving an invitation to participate in the HPP contributed to the experience. Depending on previous experiences, the confirmation of human values could both disprove feelings of not being considered and strengthen previous feelings of being a valuable person or citizen:

But at the same time I feel a bit [laughs], and not just because I moved to Sweden, a bit left out. I have my background from Finland and my situation, from the war and everything. So that [short pause], yes, I thought, that now, isn't it good that they want us to feel well. Just as good as the Swedes. (Participant 2)

*Confirming abilities* means to acknowledge older persons’ ability to act to influence their situation. Confirmation is experienced as being shown that it is possible to influence life despite old age. This key process is supported by actions that lead to a confirmation of previous actions, current abilities, and prerequisites in the home environment as well as possible improvements for the future. In this, the process opens doors to awareness either by showing that one is on the right path or by convincing participants that it is possible to make changes. The key process is being shaped by the group by enabling comparison with peers and dialogue with personnel. Another action that could confirm participants’ abilities is the possibility of testing their function. The importance of testing one's function to confirm abilities and thereby becoming aware of capabilities was described by one participant:

You have to adapt to conditions in your own life; you don't dare to take on major challenges before you've understood that you can get to the next step. Thus, I think that (the tests) are incredibly important so it was lucky that [name of staff member] came along. (Participant 6)

## Discussion

The main finding in this study was the visualization of how participants and personnel jointly shaped three different key processes to health promotion: *enabling community*, *providing opportunities to understand and be understood*, and *confirming human values and abilities*. Therefore, participants experienced the HPP content and design as promoting health by *opening doors jointly to awareness about past, present, and later life*. Thus, the present findings deepen the understanding of these key processes and their meaning in health promotion for persons aging in the context of migration. This contributes to the scientific knowledge base by understanding what is in the black box of the HPP.

The category *being shaped by the group* showed that the group was central to understanding which health-promoting messages were implemented. Our results visualized how the group shaped the contextual prerequisites to implement health-promoting messages or actions. The context in which an intervention is implemented is described as an influential determinant of the success or failure to get evidence to practice (Graham et al., 2006; Rycroft-Malone, 2004). The concept of *context* can be understood in different ways, from the characteristics of the settings where changes are taking place to the theoretical underpinnings of the intervention being implemented (Damschroder et al., 2009). The theoretical underpinnings of program components such as the person-centered approach and peer learning contribute to viewing the participants as people with expertise and resources to share knowledge (Ekman et al., 2011; Shiner, 1999). The person-centered approach also highlights the establishment of a partnership that means that both personnel and participants are seen as experts (Ekman et al., 2011). This may explain the experiences of being able to shape the content and design according to one's experienced needs, while at the same time being influenced jointly by personnel and other participants.

The overarching goal of the Promoting Aging Migrants’ Capabilities program was to enable the participants to gain strategies to solve various problems that may arise at home so that they can remain living at home safely and securely (Gustafsson et al., 2015). The key processes, *providing opportunities to understand and be understood* and *confirming human values and abilities*, are in line with this goal. Both processes supported the ability of participants to become aware of health-promoting messages, to see themselves as valuable and capable persons, and the prerequisites to age in place. In contrast, the key process *enabling community* was not related to learning and the opportunity to interact with other people was sometimes the strongest motivation for participation. The meaning of community for experiencing health or choosing to participate in health-promoting activities is supported by previous findings that describe the importance of meeting others with similar experiences (Capalb, O'Halloran, & Liamputtong, 2014; Lood, Häggblom-Kronlöf, & Dellenborg, 2015; Povlsen, 2012). This suggests that the HPP can be seen as both the means and goal for meaningful occupation. Therefore, group meetings could facilitate health promotion that aimed to support meaningful occupation.

The evidence for group-based health promotion for older people is growing because of promising results, and learning from peers has been shown to be beneficial (Behm et al., 2013, 2014; Clark et al., 2001; Eklund, Sjöstrand, & Dahlin-Ivanoff, 2008; Gustafsson et al., 2012). For example, group-based health promotion has been described as offering a supportive environment for learning by enabling interactions with peers in similar situations (Behm et al., 2013). The advantage of peer learning is confirmed in each of the three key processes. However, the key process *providing opportunities to understand and be understood* highlights the importance of being able to choose how one wishes to exchange knowledge, with whom, and about what. The ability to choose facilitated the opening of doors to awareness because experiential barriers could be avoided and environmental demands adapted to fit individual interests, needs, and resources. A person's effective possibilities to convert resources into the achievement of a goal that he or she has a reason to value is referred to as *capability* and the degree of capability is related to environmental conditions (Sen, 1993). Therefore, the HPP seemed to improve capability because the learning environment enabled choices that supported persons’ conversion of resources into health. Our results suggest that prerequisites to choose emerged because of the multidimensional, bilingual content, variety in pedagogical approaches, and provision of an interdisciplinary team where the participants contributed with their expertise as peers.

Previous studies have shown that it is common for older people to dismiss thoughts about the future and to live in the “present” instead (Behm et al., 2013). The ways narratives can contribute to supporting persons in becoming aware about the past, present, and later life have been described previously. A study conducted by Glass, Moss, and Ogle (2012) shows that sharing stories serves as a map of where we have been, where we are, and where we might go. Our results suggest that receiving information or questions that would normally not be considered enabled the participants to come in touch with their past, present, and later life issues. Therefore, both personnel and participants helped open the doors to awareness of past, present, and later life through their different roles and expertise. However, the results also illustrate that there might be obstacles to implementing health-promoting messages because of the composition of groups. Therefore, group composition is crucial and should be considered in HPPs to facilitate the process of opening doors jointly to awareness of past, present, and later life.

### Methodological considerations and limitations

Translated data are always a limitation (Squires, 2008), but at the same time it is a prerequisite to be able to include data from participants with a mother tongue other than that of the researcher. No back translation of translated data was done. Instead translated data was validated by involving the research assistants as active partners during the analysis process. Our approach to translation is recommended in relation to the method used because both researchers and assistants were considered to contribute to the construction of meaning (Croot, Lees, & Grant, 2011; Temple, 2002). The chosen approach enabled both researchers and translators to keep a reflexive stance, which is important in improving the quality of the findings in studies with a constructivist approach (Charmaz, 2006; Temple, 2002).

Persons aging in the context of migration are a heterogeneous group of people (Torres, 2006) and initial sampling criteria were set up to maximize the heterogeneity of experiences (Hallberg, 2006). The fact that most of the included participants had migrated to Sweden at a young age from countries within Europe must be considered when interpreting the results. The age at migration may shape the experiences of aging in the context of migration (Torres, 2006), and further studies are needed to evaluate HPP from the perspective of persons who migrated later in life.

Persons with limited language skills are often excluded from research studies (Hussain-Gambles, Atkin, & Leese, 2004). To the best of our knowledge, no previous study has explored health-promoting processes and the circumstances in which these processes occur, in relation to the content and design of a person-centered HPP from the perspective of older persons aging in the context of migration. Therefore, the originality in this study refers to a deeper insight into how to facilitate the exchange of health-promoting actions and messages. These results offer implications for development, implementation, and evaluation of HPP from the perspective of older persons aging in the context of migration.

## Conclusion

It is important to include persons aging in the context of migration in the development, implementation, execution, and evaluation of HPPs. Our results visualized the benefits of jointly using the expertise of both participants and personnel to open doors to awareness. Therefore, the results give clues as to how content and design of HPPs could facilitate or hinder the implementation of health-promoting messages. The provision of choices to meet the participants’ interests, needs, and resources were experienced as overcoming barriers to health promotion. Our results suggested that the prerequisites to choose emerged because of the multidimensional and bilingual content, variety in pedagogical approaches, and provision of an interdisciplinary team where the participants contributed with their expertise as peers. Therefore, the study provided keys to enable health by deepening the understanding of facilitators and the barriers to the exchange of health-promoting messages valued by participants. This study adds new dimensions to the scientific knowledge base of how the design and content of HPPs may contribute to support and recognize the capabilities of persons aging in the context of migration.
